# Three new species of picobiine mites (Acari: Syringophilidae) parasitising African flycatchers (Aves: Muscicapidae)

**DOI:** 10.1007/s11230-012-9376-5

**Published:** 2012-09-15

**Authors:** Maciej Skoracki, Piotr Solarczyk, Bozena Sikora

**Affiliations:** 1 Department of Animal Morphology, Faculty of Biology, Adam Mickiewicz University, Umultowska 89, 61-614 Poznan, Poland; 2 Department of Biology and Medical Parasitology, Faculty of Medicine I, Poznan University of Medical Sciences, 10 Fredry Street, 61-701 Poznan, Poland

## Abstract

Three new species of quill mites of the subfamily Picobiinae Johnston & Kethley, 1973 (Acari: Syringophilidae) are described from African flycatchers (Passeriformes: Muscicapidae): *Picobia cichladusa* n. sp. on *Cichladusa arquata* Peters and *P. myrmecocichla* n. sp. on *Myrmecocichla arnotti* (Tristram), both from Tanzania, and *P. echo* n. sp. on *Cossypha heuglini* Hartlaub from the Democratic Republic of the Congo.

## Introduction

The family Syringophilidae Lavoipierre, 1953 includes highly specialised parasites of birds. These mites live and reproduce inside the quills of various feather types (e.g. primaries, secondaries, coverts, rectrices and body feathers), feeding on the soft tissue fluids of their hosts by piercing the calamus wall with their long and flexible cheliceral digits (Kethley, 1971). The host-parasite relationships of syringophilids and their biodiversity are still poorly known. To date the family includes 269 species of 53 genera recorded from more than 180 bird species belonging to 69 families of 21 orders from all of the geographical regions, except for the Antarctic (Skoracki, 2011; Skoracki & OConnor, 2010; Skoracki et al., 2011, 2012). However, the actual number of syringophilid species is estimated to be at least 5,000 based on the species numbers of their potential hosts (Johnston & Kethley, 1973). In contrast to members of the subfamily Syringophilinae Lavoipierre, 1953, which mainly inhabit the flight feathers, most species of the subfamily Picobiinae Johnston & Kethley, 1973, except for species of *Calamincola* Casto, 1978, occupy quills of the body feathers. This difference is likely a result of a basal divergence at an early stage of syringophilid evolution (Skoracki et al., 2004).

The subfamily Picobiinae includes 36 species of five genera (excluding three species assigned as *incertae sedis*): these include *Picobia* Haller, 1878 with 19 species parasitising birds of the orders Passeriformes, Piciformes and Upupiformes; *Rafapicobia* Skoracki, 2011 with two species found on passerines; *Neopicobia* Skoracki, 2011 with 10 species found on Passeriformes, Columbiformes and Psittaciformes; *Columbiphilus* Kivganov & Sharafat, 1995 with four species, one known from a columbiform host and the remaining species from galliforms; and *Calamincola* Casto, 1977 with a single species described from a cuculiform host (Casto, 1977; Kivganov & Sharafat, 1995; Fain et al., 2000; Skoracki et al., 2004; Skoracki, 2011; Skoracki & Sikora, 2011; Sikora et al., 2011; Glowska & Skoracki, 2011; Glowska et al., 2011).

To date, only one picobiine species, *Rafapicobia zinitra* Skoracki, 2011, has been described from muscicapid hosts, i.e. *Saxicola rubetra* (Linnaeus) and *Ficedula hypoleuca* (Pallas). In this paper, we describe three new species of *Picobia* found on African flycatchers.

## Materials and methods

The material used in the present study was collected from dry bird skins housed in the ornithological collection of the Bavarian State Collection of Zoology (ZSM), Munich, Germany. Mites were extracted with sharp, fine tweezers through a longitudinal cut made in the quill. Before mounting, mites were softened and cleared in 10% lactic acid at 60°C for 2–3 days. For light microscope studies, mites were mounted on slides in Faure’s medium and examined under an Olympus BH-2 light microscope with differential interference contrast (DIC) optics. Drawings were made with camera lucida. All measurements, including the figure scale-bars, are given in micrometres. The idiosomal setation follows Grandjean (1939), as adapted for the Prostigmata by Kethley (1990). The system of nomenclature for leg setation follows that proposed by Grandjean (1944), as adapted for the Syringophilidae by Bochkov et al. (2008) and Skoracki (2011). Bird taxonomy and nomenclature of birds follow Clements (2007). Specimen depositories and reference numbers are cited using the following abbreviations: AMU, A. Mickiewicz University, Natural History Collection, Poznan, Poland; ZSM, Bavarian State Collection of Zoology, Munich, Germany.

**Family Syringophilidae Lavoipierre, 1953**

**Subfamily Picobiinae Johnston & Kethley, 1973**

**Genus** ***Picobia*** **Haller, 1878**

## *Picobia cichladusa* n. sp.

*Type-host*: *Cichladusa arquata* Peters (Passeriformes: Muscicapidae).

*Type-locality*: Tanzania, Soga, 16 July 1960, coll. Th. Andersen.

*Type-material*: Female holotype (non-physogastric form) and paratypes: 1 female (non-physogastric form), 2 females (physogastric form), 1 male, 2 nymphs. Mites removed by M. Skoracki. Host specimen deposited in the ZSM. All type-material is deposited in the ZSM (Reg. No. ZSM 20112002), except for 1 female paratype (physogastric form) in the AMU (Reg. No. AMU-SYR.371).

*Etymology*: The name of this species refers to the generic name of the host.

### Description (Figs. 1–9)

**Figs. 1, 2:**
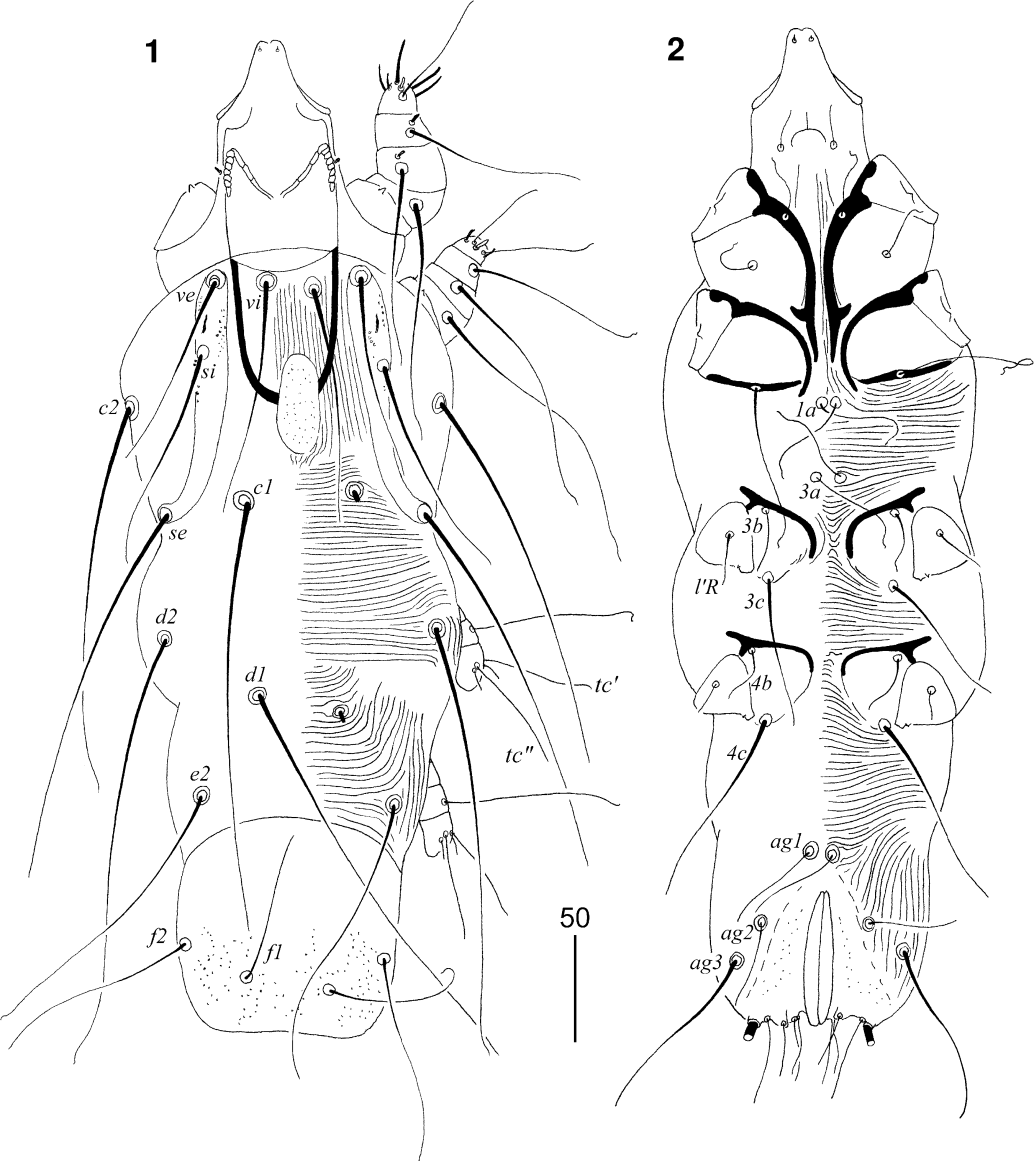
*Picobia cichladusa* n. sp., female: 1, dorsal view; 2, ventral view

**Figs. 3–7:**
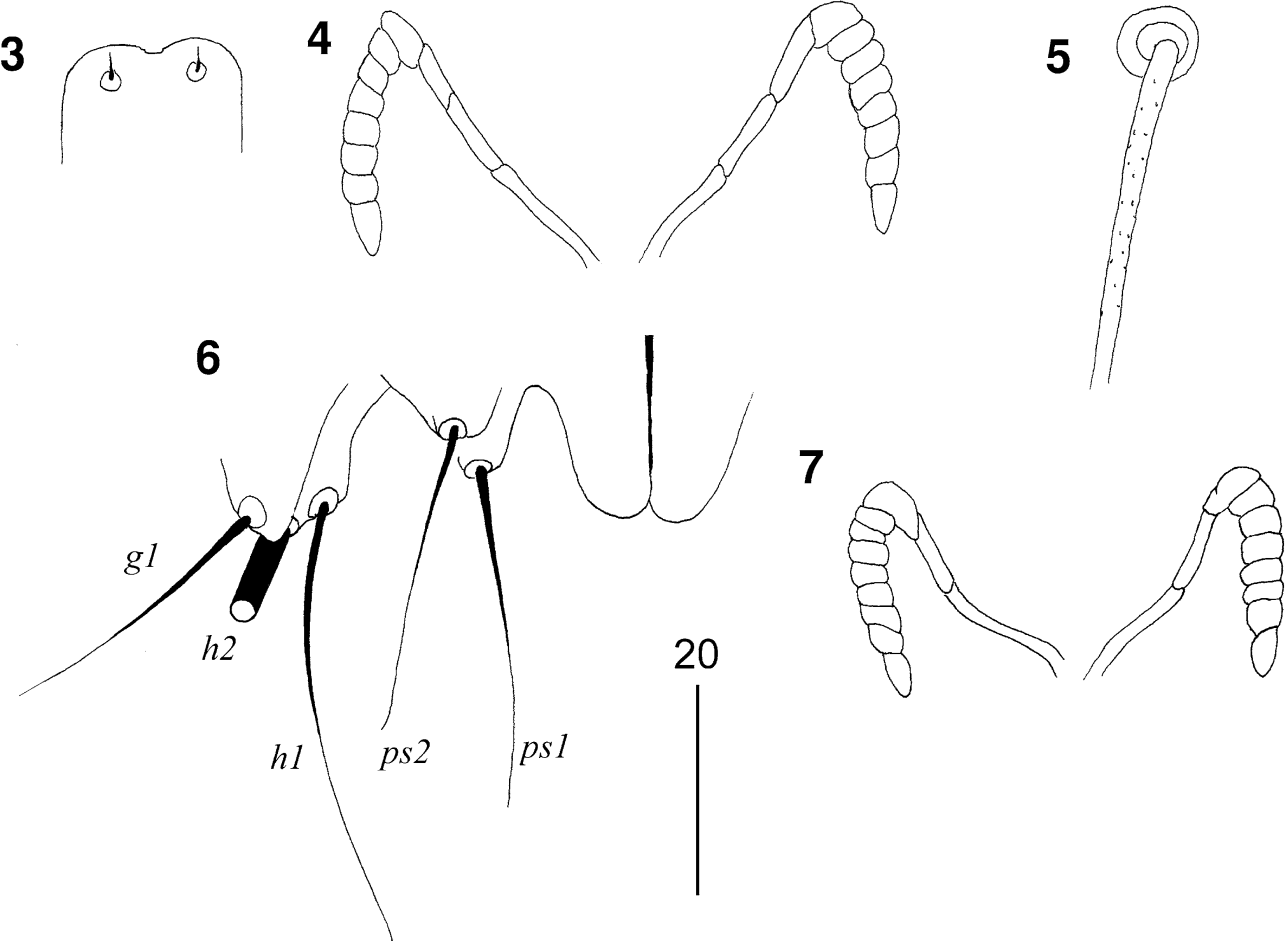
*Picobia cichladusa* n. sp.: (3–6), female: 3, hypostomal apex; 4, peritremes; 5, propodonotal seta *ve*; 6, terminal opisthosoma in ventral view; (7), male: 7, peritremes

*Non-physogastric female* (Figs. 1–5). [Based on holotype and 1 paratype.] Total body length 485 (555). *Gnathosoma*. Hypostomal apex rounded, without shoulders (Fig. 3). Infracapitulum apunctate. Movable cheliceral digit edentate posteriorly, 145 (145) long. Each medial branch of peritremes with 3–4 chambers; each lateral branch with 7 chambers (Fig. 4). Stylophore 185 (180) long. Podomers of palps densely punctate. *Idiosoma*. Propodonotal shield punctate, divided into 2 narrow lateral and single oval median shields. Length ratio of setae *vi:ve:si* is 1.3:1:1.3. Bases of setae *vi* and *ve* situated at same transverse level. Setae *c1* located slightly anterior to level of setae *se*. Dorsal setae of idiosoma and legs lightly beaded (Fig. 5). Pygidial shield well developed, punctate. Setae *f2* 1.4 times longer than *f1*. Setae *f1* 1.4–1.5 times longer than *h1*. Aggenital setae *ag1* situated anterior to level of setae *ag2*. Length ratio of setae *ag1*:*ag2*:*ag3* 1.3:1:2. Genital plate present, punctate. Genital lobes present. Pseudanal setae *ps1* and *ps2* subequal in length. Genital setae filiform, situated on genital lobes, subequal to pseudanal setae (Fig. 6). All coxal fields well sclerotised, apunctate. Setae *4c* 1.3 times longer than *3c*. Length ratios of setae *3b:3c* and *4b:4c* 1:2.3 and 1:3.2, respectively. *Legs*. Antaxial and paraxial members of claws subequal in size. Setae *tc*′ and *tc*″ of legs III–IV subequal in length. *Lengths of setae*: *vi* 115, *ve* 90 (90), *si* 115 (120), *se* 210 (180), *c1* 220 (215), *c2* 195 (180), *d1* 205 (180), *d2* 210 (195), *e2* 150 (155), *f1* 70 (65), *f2* 100 (95), *h1* 50, *h2* >200, *ag1* 55 (60), *ag2* 40 (45), *ag3* 85 (90), *g1* 30 (30), *ps1* 30 (30), *ps2* 30 (30), *tc*′*III–IV* 50 (55), *tc*″*III–IV* 50 (55), *l*′*RIII* 30 (25), *3b* (30), *3c* 70 (65), *4b* 30 (30), *4c* 95 (95).

*Physogastric female*. [Based on 1 paratype.] Body, vermiform in outline, 895 long. Other characters as in non-physogastric form.

**Figs. 8, 9:**
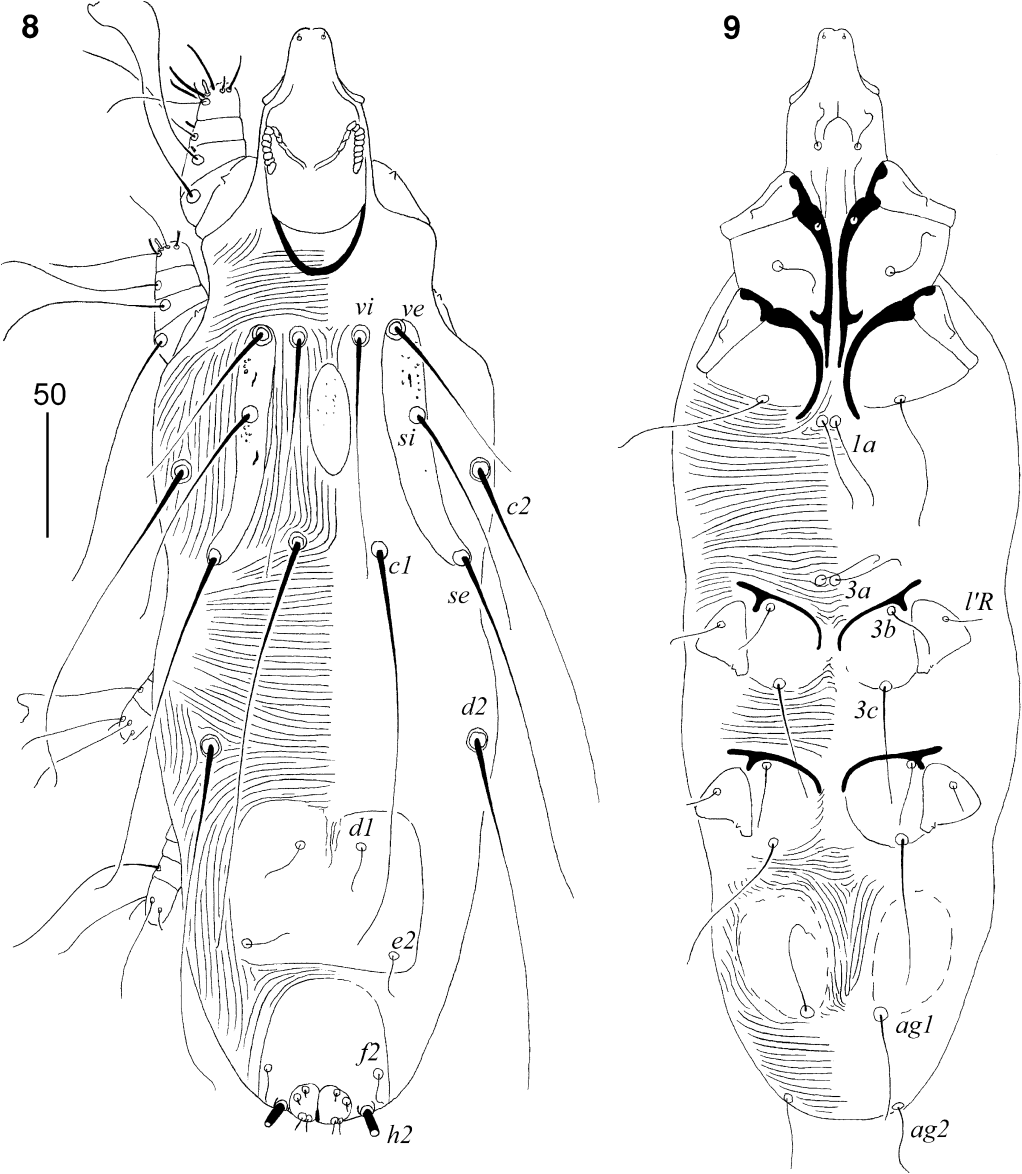
*Picobia cichladusa* n. sp., male: 8, dorsal view; 9, ventral view

*Male* (Figs. 7–9). [Based on 1 paratype.] Total body length 435. *Gnathosoma*. Hypostomal apex rounded. Infracapitulum apunctate. Stylophore 100 long. Each medial branch of peritremes with 3 chambers; each lateral branch with 7–8 chambers (Fig. 7). *Idiosoma*. Propodonotal shield divided into 2 narrow and sparsely punctate lateral shields and unpaired oval shield situated in middle of propodonotum. Length ratio of setae *vi:ve:si* are 1.3:1:1.3. Idiosomal setae *vi*, *ve*, *si*, *se*, *c1*, *c2* and *d2* slightly beaded; other idiosomal setae smooth. Bases of setae *vi* and *ve* situated at same transverse level. Setae *c1* and *se* situated at same transverse level. Hysteronotal shield well developed, entire, apunctate, bearing bases of setae *d1* and *e2*. Setae *d2* about 9 times longer than *d1* and *e2*. Pygidial shield well sclerotised, apunctate. Setae *h2* about 15 times longer than *f2*. Two aggenital plates present, bearing bases of setae *ag1* on posterior margin. Setae *ag1* 1.8 times longer than *ag2*. All coxal fields well sclerotised and apunctate. Setae *4c* 1.4 times longer than *3c*. Length ratios of setae *3b:3c* and *4b:4c* 1:2.3 and 1:2.6, respectively. *Legs*. Dorsal setae of all legs slightly beaded. Setae *tc*′ and *tc*″ of legs III–IV subequal in length. *Lengths of setae*: *vi* 100, *ve* 75, *si* 100, *se* 140, *c1* 165, *c2* 130, *d1* 15, *d2* 140, *e2* 15, *f2* 15, *h2* >140, *ag1* 45, *ag2* 25, *tc*′*III–IV* and *tc*″*III–IV* 45, *l*′*RIII* 25, *3b* 20, *3c* 45, *4b* 25, *4c* 65.

### Differential diagnosis

*Picobia cichladusa* n. sp. is morphologically similar to *P. currucae* Skoracki & Magowski, 2001 described from *Sylvia curruca* (Linnaeus) (Passeriformes: Sylviidae) in Poland (Skoracki & Magowski, 2001). In females of both species: the hypostomal apex is rounded; the peritremes are M-shaped; the movable cheliceral digit is edentate posteriorly; the pygidial and genital shields are well developed; the genital setae are filiform and situated on well developed genital lobes; and the hysteronotal is absent. This new species differs from *P. currucae* by the following characters: in females of *P. cichladusa*, aggenital setae *ag2* are situated posterior to the level of setae *ag1*; genital setae *g1* and pseudanal setae *ps1* and *ps2* are subequal in length; the propodonotal shield is punctate and divided into two narrow lateral shields and a single oval median shield. Whereas, in females of *P. currucae*, aggenital setae *ag1* and *ag2* are situated at the same transverse level; genital setae *g1* are 2–2.5 times longer than pseudanal setae *ps1* and *ps2*; and the propodonotal shield is apunctate and divided only into two narrow lateral shields, i.e. the unpaired median shield is absent.

## *Picobia echo* n. sp.

*Type-host*: *Cossypha heuglini* Hartlaub (Passeriformes: Muscicapidae).

*Type-locality*: D. R. Congo, South Kivu Prov., Bukavu, 7 August 1969, coll. P. Kunkel.

*Type-material*: Female holotype (non-physogastric form) and paratypes: 4 females (physogastric form), 2 males, 2 nymphs and 1 larva. Host specimen is deposited in the ZSM. Mites removed by M. Skoracki. All material is deposited in the ZSM (Reg. No. ZSM 20112010), except 1 female paratype (physogastric form) in the AMU (Reg. No. AMU-SYR.372).

*Etymology*: The name of this species derives from the Greek εχώ (*ēchō*), “sound”.

### Description (Figs. 10–21)

**Figs. 10, 11:**
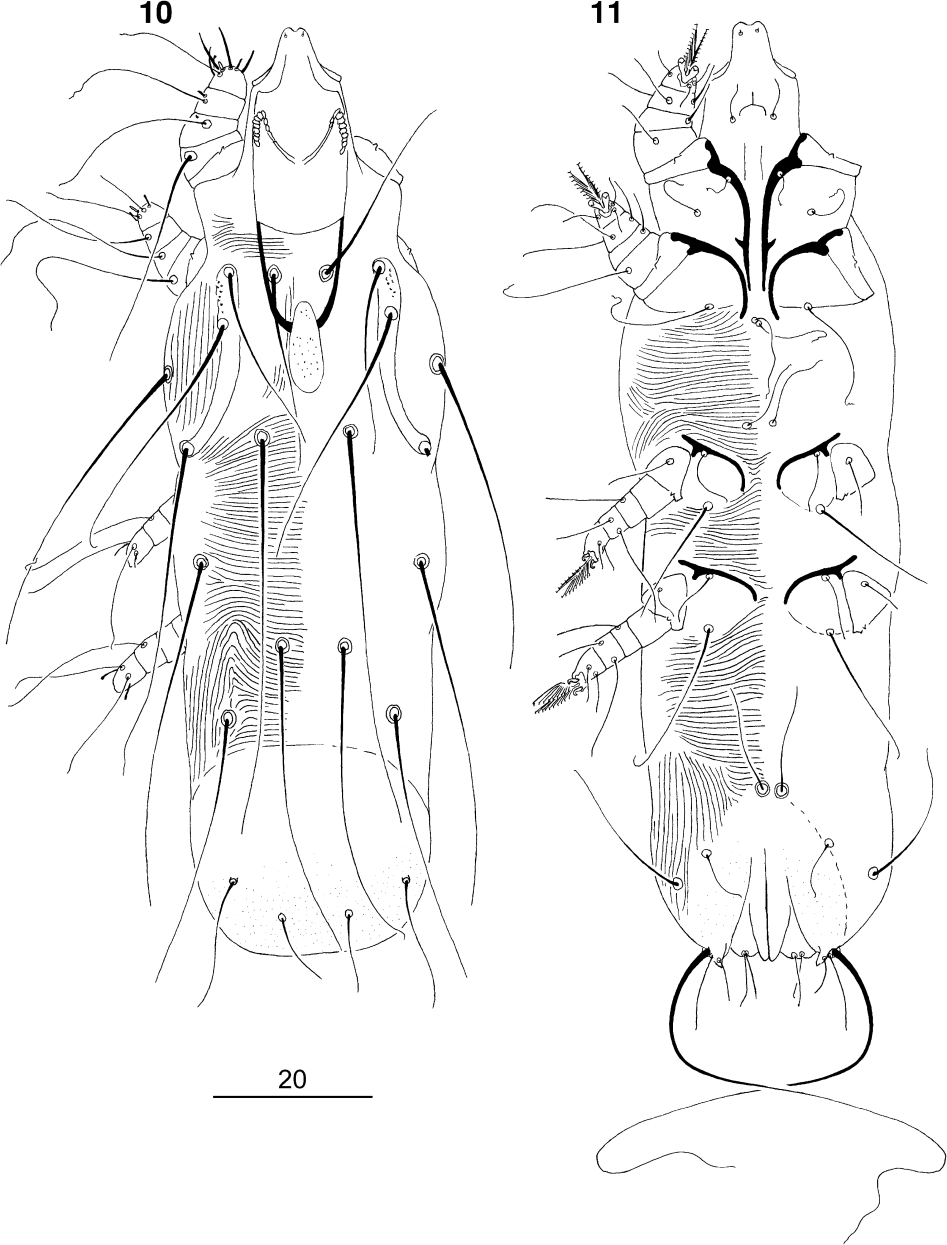
*Picobia echo* n. sp., female: 10, dorsal view; 11, ventral view

**Figs. 12–19:**
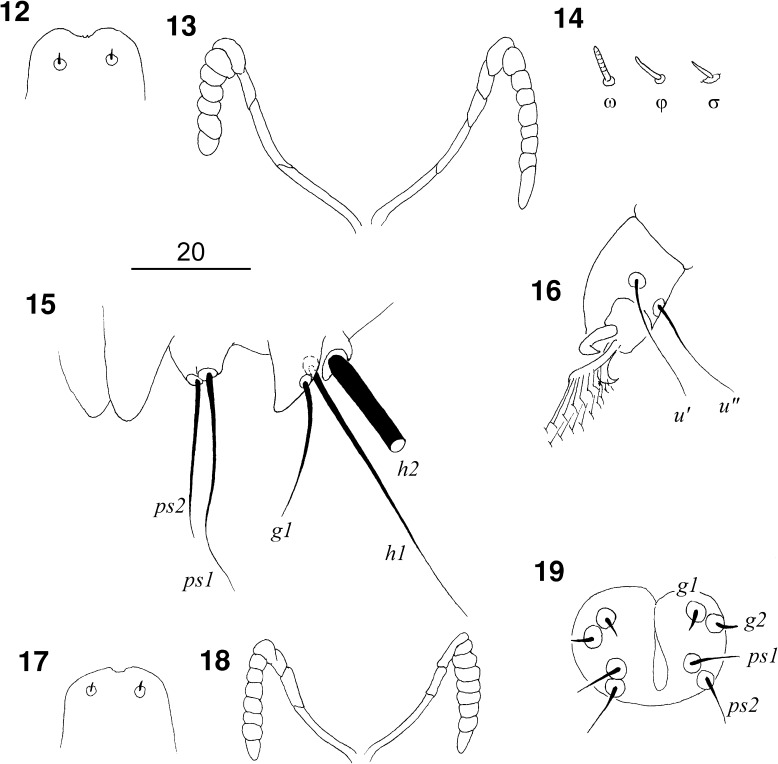
*Picobia echo* n. sp.: (12–16) female: 12, hypostomal apex; 13, peritremes; 14, solenidia of leg I; 15, terminal opisthosoma in ventral view; 16, tarsus III in ventral view; (17–19) male: 17, hypostomal apex; 18, peritremes; 19, genito-anal region

*Non-physogastric female* (Figs. 10–16). [Based on holotype.] Total body length 565. *Gnathosoma*. Hypostomal apex rounded, without shoulders (Fig. 12). Infracapitulum apunctate. Movable cheliceral digit edentate posteriorly, 145 long. Each medial branch of peritremes with 4–5 chambers; each lateral branch with 6–8 chambers (Fig. 13). Stylophore 185 long. Podomers of palps densely punctate. *Idiosoma*. Propodonotal shield punctate, divided into 2 narrow lateral and single oval median shields. Length ratio of setae *vi:ve:si* is 1:1:1.4. Bases of setae *vi* and *ve* situated at same transverse level. Setae *c1* set slightly anterior to level of setae *se*. Dorsal setae of idiosoma (except terminal setae *h2*) lightly beaded. Pygidial shield well developed, punctate. Setae *f2* twice as long as *f1*. Setae *f1* and *h1* subequal in length. Aggenital setae *ag1* situated anterior to level of setae *ag2*. Length ratio of setae *ag1:ag2:ag3* 1.4:1:2. Genital plate present, punctate. Genital lobes present. Pseudanal setae *ps1* 1.4 times longer than *ps2*. Genital setae filiform, situated on genital lobes (Fig. 15). All coxal fields well sclerotised, apunctate. Setae *4c* not significant (1.1–1.2 times) longer than *3c*. Length ratios of setae *3b:3c* and *4b:4c* 1:2.4. *Legs*. Antaxial and paraxial members of claws subequal in size (Fig. 16). Setae *tc*′ and *tc*″ of legs III–IV subequal in length. Dorsal setae of all legs lightly beaded. *Lengths of setae*: *vi* 125, *ve* 125, *si* 180, *se* 215, *c1* 240, *c2* 205, *d1* 190, *d2* 220, *e2* 185, *f1* 45, *f2* 90, *h1* 45, *h2* >200, *ag1* 65, *ag2* 45, *ag3* 90, *g1* 20, *ps1* 35, *ps2* 25, *tc*′*III–IV* 60, *tc*″*III–IV* 60, *l*′*RIII* 35, *3b* 35, *3c* 85, *4b* 40, *4c* 95.

*Physogastric female*. [Based on 1 paratype.] Body, vermiform outline, 1,100 long. Other characters as in non-physogastric form.

**Figs. 20–21:**
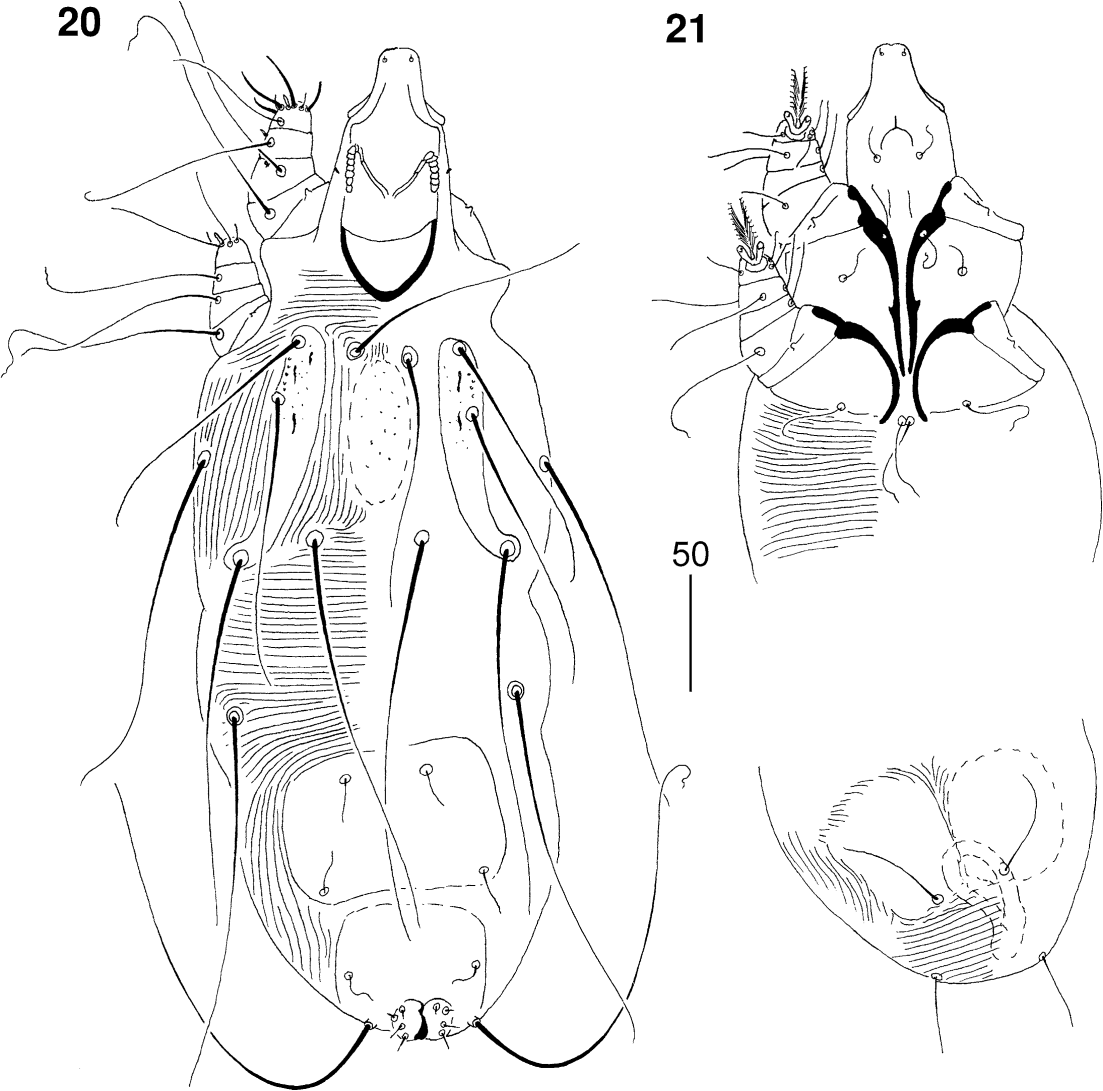
*Picobia echo* n. sp., male: 20, dorsal view; 21, propodosoma and opisthosoma in ventral view

*Male* (Figs. 17–21). [Based on 2 paratypes.] Total body length 380–410. *Gnathosoma*. Hypostomal apex rounded (Fig. 17). Infracapitulum apunctate. Stylophore 105 long. Each medial branch of peritremes with 3–4 chambers; each lateral branch with 7–8 chambers (Fig. 18). *Idiosoma*. Propodonotal shield punctate, divided into 2 narrow lateral shields and one unpaired oval shield situated in middle of propodonotum. Length ratio of setae *vi:ve:si* 1:1:1.1–1.2. Idiosomal setae *vi*, *ve*, *si*, *se*, *c1*, *c2* and *d2* slightly beaded; other setae smooth. Bases of setae *vi* and *ve* situated at same transverse level. Setae *c1* situated slightly anterior to level of setae *se*. Hysteronotal shield well developed, entire, apunctate, bearing bases of setae *d1* and *e2*. Setae *d2* about 7 times longer than *d1* and *e2*. Pygidial shield well sclerotised, apunctate. Setae *h2* about 13 times longer than *f2*. Two aggenital plates present, situated close to each other, bearing bases of setae *ag1*. Setae *ag1* 1.3–1.8 times longer than *ag2*. All coxal fields well sclerotised and apunctate. Setae *4c* not significant (1.1–1.2 times) longer than *3c*. *Legs*. Dorsal setae of all legs slightly beaded. Setae *tc*′ and *tc*″ of legs III–IV subequal in length. *Lengths of setae*: *vi* 105, *ve* 100–115, *si* 100–125, *se* 155–160, *c1* 165, *c2* 150–160, *d1* 20, *d2* 140, *e2* 20, *f2* 15, *h2* 160–190, *ag1* 45, *ag2* 25–35, *tc*′*III–IV* and *tc*″*III–IV* 50–55, *l*′*RIII* 25, *3b* 25, *3c* 50, *4b* 25, *4c* 55–60.

### Differential diagnosis

This species is morphologically similar to *P. cichladusa* n. sp., described above, and is distinguished by the following characters: in females of *P. cichladusa*, setae *vi* are longer than *ve* (1.3 times); setae *si* and *vi* are subequal in length; the length ratio of setae *f1:f2* is 1:1.4; and the lengths of setae *se*, *e2* and *f1* are 115–120, 150–155 and 60–70 μm, respectively. In females of *P. echo* n. sp., setae *vi* and *ve* are subequal in length; setae *si* are 1.4 times longer than *vi*; the length ratio of setae *f1:f2* is 1:2; and the lengths of setae *se*, *e2* and *f1* are 180, 185 and 45 μm, respectively.

## *Picobia myrmecocichla* n. sp.

*Type-host*: *Myrmecocichla arnotti* (Tristram) (Passeriformes: Muscicapidae).

*Type-locality*: Tanzania, 10 September 1963, coll. Th. Andersen.

*Type-material*: Female holotype (non-physogastric form) and paratypes: 1 female (non-physogastric form), 2 females (physogastric form), 2 males, 1 nymph. Mites removed by M. Skoracki. Host specimen is deposited in the ZSM. All material is deposited in the ZSM (Reg. No. ZSM 20112011), except 1 female paratype (physogastric form) in the AMU (Reg. No. AMU-SYR.375).

*Etymology*: The name of this species refers to the generic name of the host.

### Description (Figs. 22–32)

**Figs. 22, 23:**
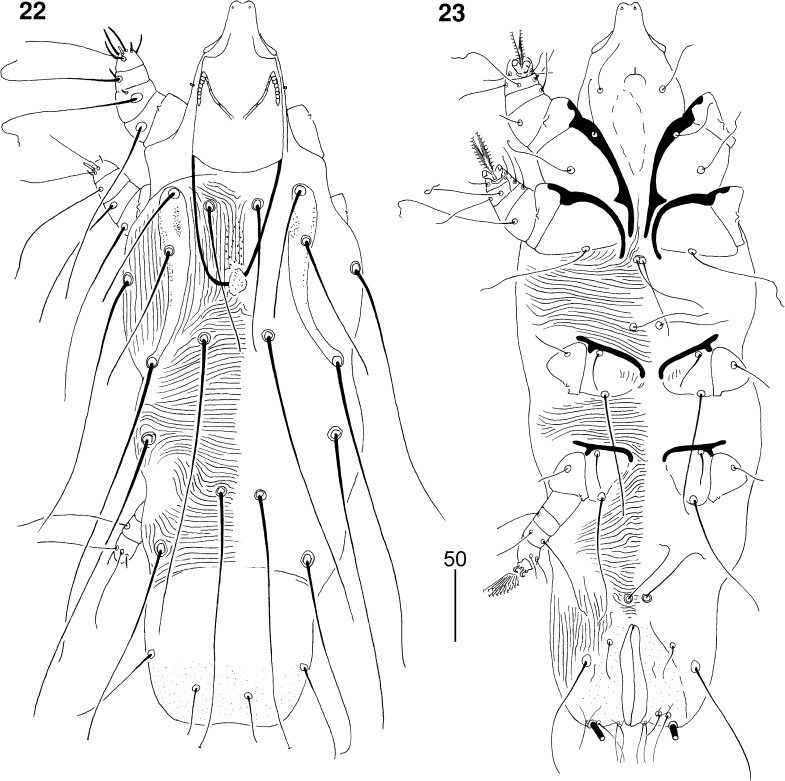
*Picobia myrmecocichla* n. sp., female: 22, dorsal view; 23, ventral view

**Figs. 24–30:**
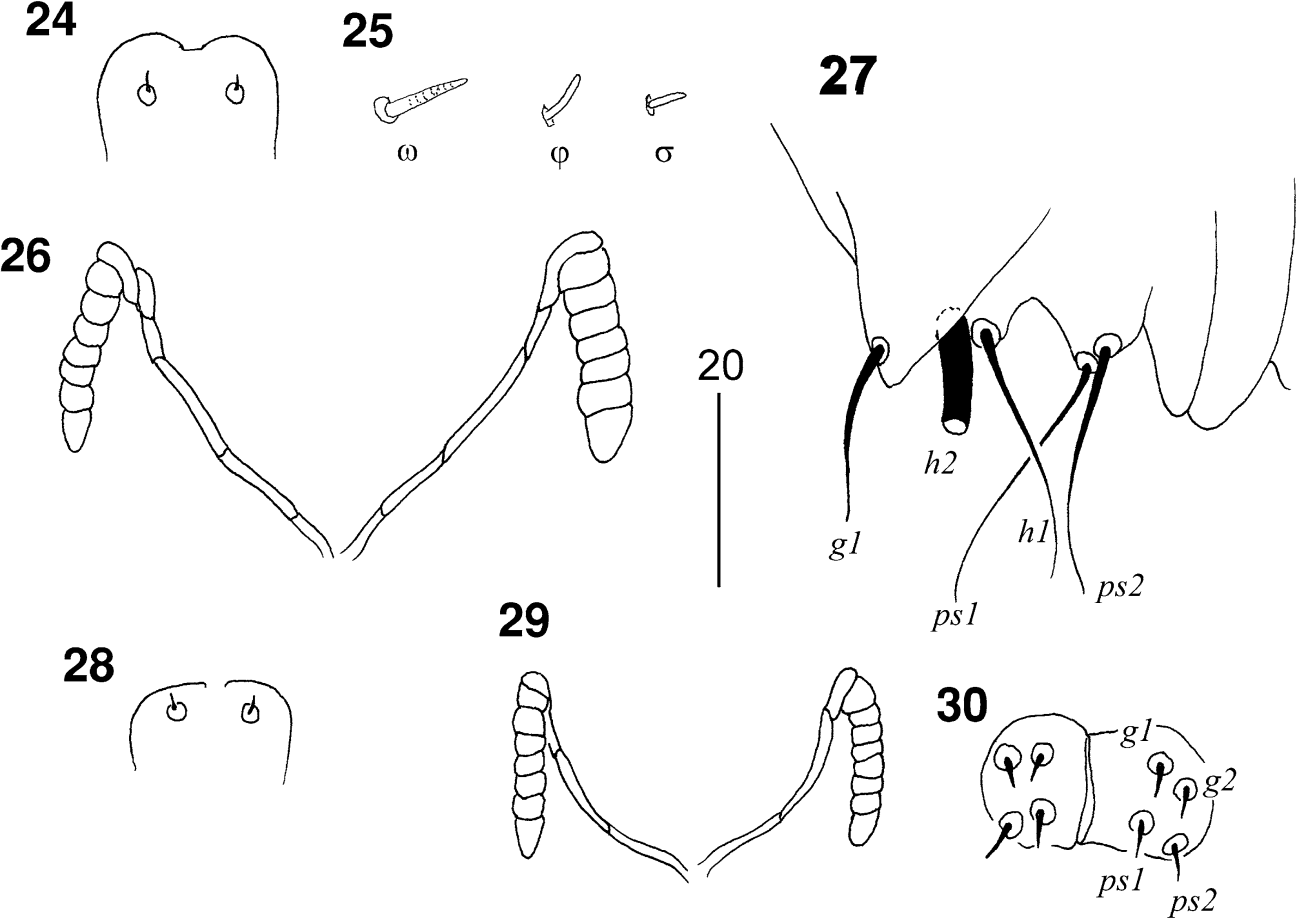
*Picobia myrmecocichla* n. sp.: (24–27) female: 24, hypostomal apex; 25, solenidia of leg I; 26, peritremes; 27, terminal opisthosoma in ventral view; (28–30) male: 28, hypostomal apex; 29, peritremes; 30, genito-anal region

*Non-physogastric female* (Figs. 22–27). [Based on holotype and 1 paratype.] Total body length 530 (520). *Gnathosoma*. Hypostomal apex rounded, without shoulders (Fig. 24). Infracapitulum apunctate. Movable cheliceral digit edentate posteriorly, 160 (160) long. Each medial branch of peritremes with 5–6 chambers; each lateral branch with 7 chambers (Fig. 26). Stylophore 200 (195) long. Podomers of palps densely punctate. *Idiosoma*. Propodonotal shield divided into 2 narrow and punctate lateral shields; median shield reduced to small sclerite or absent. Length ratio of setae *vi:ve:si* is 1.4–1.6:1:1.4–1.6. Bases of setae *vi* situated slightly posterior to level of setae *ve*. Setae *c1* located anterior to level of setae *se*. Dorsal setae of idiosoma and legs lightly beaded. Pygidial shield well developed, punctate. Setae *f2* 2–2.3 times longer than *f1*. Setae *f1* and *h1* subequal in length. Aggenital setae *ag1* situated anterior to level of setae *ag2*. Length ratio of setae *ag1:ag2:ag3* 2:1:2.7. Genital plate punctate. Genital lobes present. Pseudanal setae *ps1* and *ps2* subequal in length. Genital setae filiform, situated on genital lobes. All coxal fields well sclerotised, apunctate. Setae *4c* slightly (1.1–1.2 times) longer than *3c*. Length ratios of setae *3b:3c* 1:2.5–2.7. *Legs*. Antaxial and paraxial members of claws subequal in size. Setae *tc*′ and *tc*″ of legs III–IV subequal in length. *Lengths of setae*: *vi* 110 (125), *ve* 80 (80), *si* 110 (125), *se* 215 (220), *c1* 235 (215), *c2* (205), *d2* 225 (225), *e2* 145 (140), *f1* 35 (30), *f2* 70 (70), *h1* 35 (30), *h2* >200, *ag1* 55 (60), *ag2* (30), *ag3* 75 (80), *g1* 25, *ps1* (30), *ps2* 25, *tc*′*III–IV* 55 (55), *tc*″*III–IV* 55 (55), *l*′*RIII* 30 (25), *3b* 30 (30), *3c* 75 (80), 4*c* 90 (90).

*Physogastric female*. [Based on 1 paratype.] Body, vermiform outline, 1,050 long. Other characters, excluding pygidial shield clearly punctate, as in non-physogastric form.

**Figs. 31, 32:**
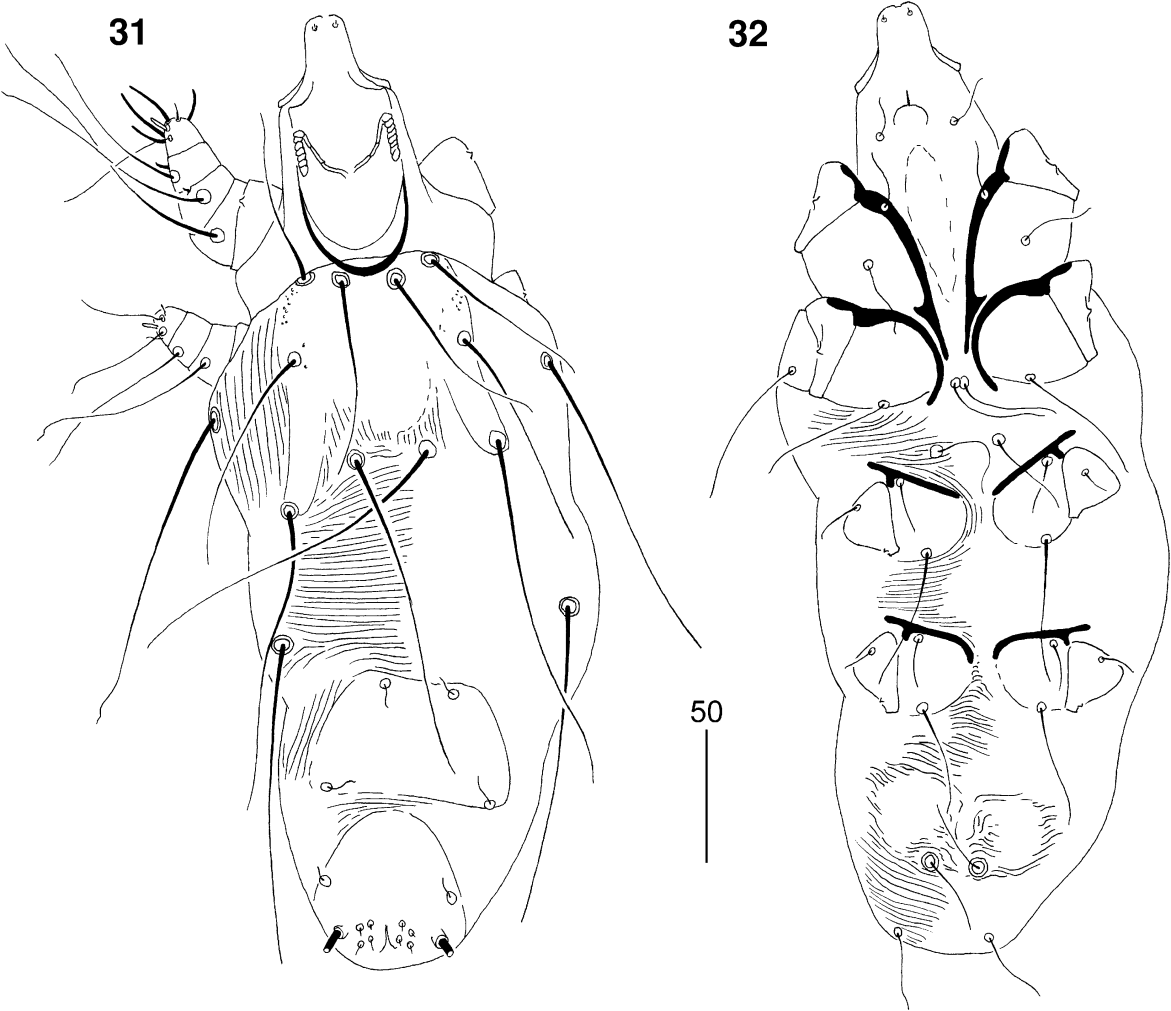
*Picobia myrmecocichla* n. sp., male: 31, dorsal view; 32, ventral view

*Male* (Figs. 28–32). [Based on 2 paratypes.] Total body length 435. *Gnathosoma*. Hypostomal apex rounded (Fig. 28). Infracapitulum apunctate. Stylophore 95–105 long. Each medial branch of peritremes with 3 chambers; each lateral branch with 7–8 chambers (Fig. 29). *Idiosoma*. Propodonotal shield not divided, long-sleeved shirt-like, punctate near bases of setae *ve* and *si*. Length ratio of setae *vi:ve:si* are 1.2–1.3:1:1.4–1.5. Idiosomal setae *vi*, *ve*, *si*, *se*, *c1*, *c2* and *d2* slightly beaded; other setae smooth. Bases of setae *vi* situated slightly posterior to level of setae *ve*. Setae *c1* situated anterior to level of setae. Hysteronotal shield well developed, trapezoidal in shape, apunctate, bearing bases of setae *d1* and *e2*. Setae *d2* 8–10 times longer than *d1* and *e2*. Pygidial shield well sclerotised, apunctate. Setae *h2* about 15 times longer than *f2*. Two aggenital plates present, weakly developed; bases of setae *ag1* situated near these plates. Setae *ag1* and *ag2* subequal in length. All coxal fields well sclerotised and apunctate. Length ratio of setae *3b:4b:3c:4c* 1:1:2.3:2–2.3. *Legs*. Dorsal setae of all legs lightly beaded. Setae *tc*′ and *tc*″ of legs III–IV subequal in length. *Lengths of setae*: *vi* 70, *ve* 55–60, *si* 80–85, *se* 140, *c1* 135, *c2* 130, *d1* 15–20, *d2* 120, *e2* 15–20, *f2* 10, *h2* 150, *ag1* 30, *ag2* 25–30, *tc*′*III–IV* and *tc*″*III–IV* 40–45, *l*′*RIII* 25, *3b* 20, *3c* 45, *4b* 20, *4c* 40.

### Differential diagnosis

This new species is morphologically similar to *P. cichladusa* n. sp., described above by the presence of setae *vi* and *si* which are subequal in length in the females. It differs from *P. cichladusa* as follows: in females of *P. myrmecocichla* n. sp., the propodonotal median shield is reduced or absent; the lengths of setae *f1* and *f2* are 30–35 and 70 μm, respectively; the length ratio of setae *ag1:ag2:ag3* is 2:1:2.7; and setae *f1* and *h1* are subequal in length; in males, the propodonotal shield is not divided; the aggenital plates are strongly reduced and setae *ag1* are situated near these plates; and setae *vi* are 70 μm long. In females of *P. cichladusa*, the propodonotal median shield is well sclerotised; the lengths of setae *f1* and *f2* are 60–70 and 95–100 μm, respectively; the length ratio of setae *ag1:ag2:ag3* is 1.3:1:2; and setae *f1* are 1.4–1.5 times longer than *h1*; in males, the propodonotal shield is divided into three sclerites; the aggenital plates are well developed and the bases of setae *ag1* are situated on these plates; and setae *vi* are 100 μm long.
